# Supplementary material for: Transcriptional Modulation of the Host Immunity Mediated by Cytokines and Transcriptional Factors in Plasmodium falciparum-Infected Patients of North-East India
Source: Biomolecules. 2019 Oct 11;9(10):600. doi: 10.3390/biom9100600 (PMC6843480; doi:10.3390/biom9100600)
Supplement: Supplementary file 1 [file biomolecules-09-00600-s001.pdf]

Table S1. Fold change analysis of cytokines and signaling molecules compared to both the Endemic and Non-Endemic healthy controls

S.N	Genes and Signaling factors	TargetΔCt	ControlΔCt	ΔΔCt	Fold Change (2-ΔΔCt)	Fold Change Reductions	Log2 fold Change	Log10 fold Change
1	IFN-gamma-UC1-EC	8.565	9.251	-0.685	1.608	NA	0.685	0.206
	IFN-gamma-UC2-EC	9.971	9.251	0.721	0.607	1.648	-0.721	-0.217
	IFN-gamma-SM-EC	10.173	9.251	0.922	0.528	1.895	-0.922	-0.278
	IFN-gamma-UC1-NEC	8.565	9.093	-0.528	1.442	NA	0.528	0.159
	IFN-gamma-UC2-NEC	9.971	9.093	0.878	0.544	1.838	-0.878	-0.264
	IFN-gamma-SM-NEC	10.173	9.093	1.080	0.473	2.114	-1.080	-0.325
2	TNF-alpha-UC1-EC	9.721	9.825	-0.104	1.075	NA	0.104	0.031
	TNF-alpha-UC2-EC	9.903	9.825	0.078	0.947	1.056	-0.078	-0.024
	TNF-alpha-SM-EC	9.358	9.825	-0.466	1.382	NA	0.466	0.140
	TNF-alpha-UC1-NEC	9.721	9.980	-0.259	1.197	NA	0.259	0.078
	TNF-alpha-UC2-NEC	9.903	9.980	-0.077	1.055	NA	0.077	0.023
	TNF-alpha-SM-NEC	9.358	9.980	-0.622	1.539	NA	0.622	0.187
3	IL10-UC1-EC	9.953	11.315	-1.362	2.571	NA	1.362	0.410
	IL10-UC2-EC	10.480	11.315	-0.835	1.784	NA	0.835	0.251
	IL10-SM-EC	9.350	11.315	-1.965	3.904	NA	1.965	0.591
	IL10-UC1-NEC	9.953	11.045	-1.092	2.132	NA	1.092	0.329
	IL10-UC2-NEC	10.480	11.045	-0.565	1.479	NA	0.565	0.170
	IL10-SM-NEC	9.350	11.045	-1.695	3.237	NA	1.695	0.510
4	TGF beta-UC1-EC	3.100	2.463	0.638	0.643	1.556	-0.638	-0.192
	TGF beta-UC2-EC	3.168	2.463	0.706	0.613	1.631	-0.706	-0.212
	TGF beta-SM-EC	3.082	2.463	0.619	0.651	1.536	-0.619	-0.186
	TGF beta-UC1-NEC	3.100	2.891	0.209	0.865	1.156	-0.209	-0.063
	TGF beta-UC2-NEC	3.168	2.891	0.277	0.825	1.212	-0.277	-0.083
	TGF beta-SM-NEC	3.082	2.891	0.191	0.876	1.141	-0.191	-0.057
5	IL12-UC1-EC	13.767	13.523	0.244	0.844	1.184	-0.244	-0.073
	IL12-UC2-EC	14.044	13.523	0.521	0.697	1.435	-0.521	-0.157
	IL12-SM-EC	14.021	13.523	0.499	0.708	1.413	-0.499	-0.150
	IL12-UC1-NEC	13.767	13.385	0.381	0.768	1.303	-0.381	-0.115
	IL12-UC2-NEC	14.044	13.385	0.658	0.634	1.578	-0.658	-0.198
	IL12-SM-NEC	14.021	13.385	0.636	0.643	1.554	-0.636	-0.191
6	GATA3-UC1-EC	11.257	10.422	0.835	0.561	1.783	-0.835	-0.251
	GATA3-UC2-EC	11.392	10.422	0.970	0.510	1.959	-0.970	-0.292
	GATA3-SM-EC	11.429	10.422	1.007	0.497	2.010	-1.007	-0.303
	GATA3-UC1-NEC	11.257	10.382	0.875	0.545	1.834	-0.875	-0.263
	GATA3-UC2-NEC	11.392	10.382	1.011	0.496	2.015	-1.011	-0.304
	GATA3-SM-NEC	11.429	10.382	1.048	0.484	2.068	-1.048	-0.315
7	TBET-UC1-EC	7.731	6.941	0.791	0.578	1.730	-0.791	-0.238
	TBET-UC2-EC	8.964	6.941	2.023	0.246	4.065	-2.023	-0.609
	TBET-SM-EC	9.187	6.941	2.247	0.211	4.745	-2.247	-0.676
	TBET-UC1-NEC	7.731	6.831	0.900	0.536	1.866	-0.900	-0.271
	TBET-UC2-NEC	8.964	6.831	2.133	0.228	4.385	-2.133	-0.642

	TBET-SM-NEC	9.187	6.831	2.356	0.195	5.120	-2.356	-0.709
8	IL4-UC1-EC	14.748	13.452	1.296	0.407	2.456	-1.296	-0.390
	IL4-UC2-EC	14.402	13.452	0.950	0.518	1.932	-0.950	-0.286
	IL4-SM-EC	14.658	13.452	1.206	0.433	2.307	-1.206	-0.363
	IL4-UC1-NEC	14.748	13.024	1.724	0.303	3.304	-1.724	-0.519
	IL4-UC2-NEC	14.402	13.024	1.378	0.385	2.598	-1.378	-0.415
	IL4-SM-NEC	14.658	13.024	1.634	0.322	3.104	-1.634	-0.492
9	IL5-UC1-EC	12.250	11.091	1.159	0.448	2.233	-1.159	-0.349
	IL5-UC2-EC	12.252	11.091	1.162	0.447	2.237	-1.162	-0.350
	IL5-SM-EC	11.740	11.091	0.649	0.638	1.569	-0.649	-0.196
	IL5-UC1-NEC	12.250	11.683	0.567	0.675	1.481	-0.567	-0.171
	IL5-UC2-NEC	12.252	11.683	0.570	0.674	1.484	-0.570	-0.171
	IL5-SM-NEC	11.740	11.683	0.057	0.961	1.041	-0.057	-0.017
10	IL13-UC1-EC	14.994	13.894	1.100	0.466	2.144	-1.100	-0.331
	IL13-UC2-EC	14.708	13.894	0.815	0.568	1.759	-0.815	-0.245
	IL13-SM-EC	14.321	13.894	0.428	0.743	1.345	-0.428	-0.129
	IL13-UC1-NEC	14.994	13.482	1.512	0.351	2.852	-1.512	-0.455
	IL13-UC2-NEC	14.708	13.482	1.227	0.427	2.340	-1.227	-0.369
	IL13-SM-NEC	14.321	13.482	0.839	0.559	1.789	-0.839	-0.253
11	IL1 beta-UC1-EC	5.190	5.168	0.022	0.985	1.015	-0.022	-0.007
	IL1 beta-UC2-EC	5.220	5.168	0.052	0.964	1.037	-0.052	-0.016
	IL1 beta-SM-EC	4.916	5.168	-0.252	1.191	NA	0.252	0.076
	IL1 beta-UC1-NEC	5.190	5.859	-0.669	1.590	NA	0.669	0.201
	IL1 beta-UC2-NEC	5.220	5.859	-0.639	1.557	NA	0.639	0.192
	IL1 beta-SM-NEC	4.916	5.859	-0.943	1.923	NA	0.943	0.284
12	EOMES-UC1-EC	8.367	6.773	1.594	0.331	3.019	-1.594	-0.480
	EOMES-UC2-EC	8.948	6.773	2.174	0.222	4.514	-2.174	-0.655
	EOMES-SM-EC	8.738	6.773	1.965	0.256	3.904	-1.965	-0.592
	EOMES-UC1-NEC	8.367	6.673	1.694	0.309	3.236	-1.694	-0.510
	EOMES-UC2-NEC	8.948	6.673	2.274	0.207	4.838	-2.274	-0.685
	EOMES-SM-NEC	8.738	6.673	2.065	0.239	4.184	-2.065	-0.622
13	CSF1-UC1-EC	8.603	7.602	1.001	0.500	2.002	-1.001	-0.301
	CSF1-UC2-EC	8.303	7.602	0.701	0.615	1.626	-0.701	-0.211
	CSF1-SM-EC	8.335	7.602	0.733	0.602	1.662	-0.733	-0.221
	CSF1-UC1-NEC	8.603	8.072	0.531	0.692	1.445	-0.531	-0.160
	CSF1-UC2-NEC	8.303	8.072	0.231	0.852	1.174	-0.231	-0.070
	CSF1-SM-NEC	8.335	8.072	0.263	0.834	1.200	-0.263	-0.079
14	PERFORIN-UC1-EC	5.783	5.250	0.533	0.691	1.447	-0.533	-0.161
	PERFORIN-UC2-EC	6.741	5.250	1.492	0.356	2.812	-1.492	-0.449
	PERFORIN-SM-EC	6.389	5.250	1.139	0.454	2.202	-1.139	-0.343
	PERFORIN-UC1-NEC	5.783	5.369	0.414	0.751	1.332	-0.414	-0.125
	PERFORIN-UC2-NEC	6.741	5.369	1.372	0.386	2.589	-1.372	-0.413
	PERFORIN-SM-NEC	6.389	5.369	1.020	0.493	2.027	-1.020	-0.307
15	GRANZYME B-UC1-EC	3.969	4.396	-0.427	1.345	NA	0.427	0.129
	GRANZYME B-UC2-EC	5.152	4.396	0.756	0.592	1.689	-0.756	-0.228
	GRANZYME B-SM-EC	4.165	4.396	-0.231	1.174	NA	0.231	0.070
	GRANZYME B-UC1-NEC	3.969	4.585	-0.616	1.533	NA	0.616	0.186

	GRANZYME B-UC2-NEC	5.152	4.585	0.567	0.675	1.482	-0.567	-0.171
	GRANZYME B-SM-NEC	4.165	4.585	-0.420	1.338	NA	0.420	0.126
16	NFκB-UC1-EC	5.831	5.728	0.103	0.931	1.074	-0.103	-0.031
	NFκB-UC2-EC	5.907	5.728	0.179	0.883	1.132	-0.179	-0.054
	NFκB-SM-EC	5.656	5.728	-0.071	1.051	NA	0.071	0.022
	NFκB-UC1-NEC	5.831	5.687	0.144	0.905	1.105	-0.144	-0.043
	NFκB-UC2-NEC	5.907	5.687	0.220	0.859	1.164	-0.220	-0.066
	NFκB-SM-NEC	5.656	5.687	-0.031	1.022	NA	0.031	0.009
17	RUNX3-UC1-EC	6.145	5.215	0.930	0.525	1.906	-0.930	-0.280
	RUNX3-UC2-EC	6.670	5.215	1.455	0.365	2.742	-1.455	-0.438
	RUNX3-SM-EC	6.260	5.215	1.045	0.485	2.063	-1.045	-0.314
	RUNX3-UC1-NEC	6.145	5.244	0.901	0.535	1.868	-0.901	-0.271
	RUNX3-UC2-NEC	6.670	5.244	1.426	0.372	2.688	-1.426	-0.429
	RUNX3-SM-NEC	6.260	5.244	1.016	0.495	2.022	-1.016	-0.306
18	RUNX1-UC1-EC	8.058	8.262	-0.204	1.152	NA	0.204	0.061
	RUNX1-UC2-EC	8.404	8.262	0.142	0.906	1.103	-0.142	-0.043
	RUNX1-SM-EC	8.977	8.262	0.715	0.609	1.641	-0.715	-0.215
	RUNX1-UC1-NEC	8.058	7.772	0.287	0.820	1.220	-0.287	-0.086
	RUNX1-UC2-NEC	8.404	7.772	0.633	0.645	1.550	-0.633	-0.190
	RUNX1-SM-NEC	8.977	7.772	1.205	0.434	2.306	-1.205	-0.363
19	STAT1-UC1-EC	4.490	5.269	-0.780	1.717	NA	0.780	0.235
	STAT1-UC2-EC	4.398	5.269	-0.872	1.830	NA	0.872	0.262
	STAT1-SM-EC	5.183	5.269	-0.086	1.062	NA	0.086	0.026
	STAT1-UC1-NEC	4.490	5.273	-0.784	1.721	NA	0.784	0.236
	STAT1-UC2-NEC	4.398	5.273	-0.876	1.835	NA	0.876	0.264
	STAT1-SM-NEC	5.183	5.273	-0.090	1.065	NA	0.090	0.027
20	STAT4-UC1-EC	7.105	5.933	1.172	0.444	2.254	-1.172	-0.353
	STAT4-UC2-EC	7.652	5.933	1.719	0.304	3.293	-1.719	-0.518
	STAT4-SM-EC	7.453	5.933	1.520	0.349	2.868	-1.520	-0.458
	STAT4-UC1-NEC	7.105	5.720	1.385	0.383	2.613	-1.385	-0.417
	STAT4-UC2-NEC	7.652	5.720	1.932	0.262	3.817	-1.932	-0.582
	STAT4-SM-NEC	7.453	5.720	1.733	0.301	3.325	-1.733	-0.522
21	STAT6-UC1-EC	3.531	2.913	0.619	0.651	1.535	-0.619	-0.186
	STAT6-UC2-EC	3.364	2.913	0.451	0.731	1.367	-0.451	-0.136
	STAT6-SM-EC	3.432	2.913	0.519	0.698	1.433	-0.519	-0.156
	STAT6-UC1-NEC	3.531	2.922	0.610	0.655	1.526	-0.610	-0.184
	STAT6-UC2-NEC	3.364	2.922	0.442	0.736	1.359	-0.442	-0.133
	STAT6-SM-NEC	3.432	2.922	0.511	0.702	1.425	-0.511	-0.154
22	IL7-UC1-EC	10.209	9.889	0.321	0.801	1.249	-0.321	-0.096
	IL7-UC2-EC	10.361	9.889	0.472	0.721	1.387	-0.472	-0.142
	IL7-SM-EC	9.486	9.889	-0.403	1.322	NA	0.403	0.121
	IL7-UC1-NEC	10.209	9.937	0.273	0.828	1.208	-0.273	-0.082
	IL7-UC2-NEC	10.361	9.937	0.424	0.745	1.341	-0.424	-0.128
	IL7-SM-NEC	9.486	9.937	-0.451	1.367	NA	0.451	0.136
23	IL12Rβ2-UC1-EC	9.735	9.570	0.165	0.892	1.121	-0.165	-0.050
	IL12Rβ2-UC2-EC	10.503	9.570	0.932	0.524	1.909	-0.932	-0.281
	IL12Rβ2-SM-EC	9.968	9.570	0.398	0.759	1.318	-0.398	-0.120

	IL12Rβ2-UC1-NEC	9.735	9.887	-0.152	1.111	NA	0.152	0.046
	IL12Rβ2-UC2-NEC	10.503	9.887	0.616	0.653	1.532	-0.616	-0.185
	IL12Rβ2-SM-NEC	9.968	9.887	0.081	0.945	1.058	-0.081	-0.024
24	CXCR3-UC1-EC	9.223	8.608	0.615	0.653	1.531	-0.615	-0.185
	CXCR3-UC2-EC	9.986	8.608	1.378	0.385	2.600	-1.378	-0.415
	CXCR3-SM-EC	9.601	8.608	0.993	0.503	1.990	-0.993	-0.299
	CXCR3-UC1-NEC	9.223	8.532	0.691	0.620	1.614	-0.691	-0.208
	CXCR3-UC2-NEC	9.986	8.532	1.454	0.365	2.739	-1.454	-0.438
	CXCR3-SM-NEC	9.601	8.532	1.068	0.477	2.097	-1.068	-0.322
25	CCR8-UC1-EC	12.295	10.758	1.537	0.345	2.901	-1.537	-0.463
	CCR8-UC2-EC	12.144	10.758	1.386	0.383	2.614	-1.386	-0.417
	CCR8-SM-EC	13.268	10.758	2.510	0.176	5.696	-2.510	-0.756
	CCR8-UC1-NEC	12.295	10.833	1.461	0.363	2.753	-1.461	-0.440
	CCR8-UC2-NEC	12.144	10.833	1.311	0.403	2.481	-1.311	-0.395
	CCR8-SM-NEC	13.268	10.833	2.435	0.185	5.406	-2.435	-0.733
26	LT alpha-UC1-EC	10.168	9.375	0.793	0.577	1.732	-0.793	-0.239
	LT alpha-UC2-EC	10.214	9.375	0.839	0.559	1.789	-0.839	-0.253
	LT alpha-SM-EC	9.915	9.375	0.540	0.688	1.454	-0.540	-0.162
	LT alpha-UC1-NEC	10.168	9.341	0.826	0.564	1.773	-0.826	-0.249
	LT alpha-UC2-NEC	10.214	9.341	0.873	0.546	1.831	-0.873	-0.263
	LT alpha-SM-NEC	9.915	9.341	0.573	0.672	1.488	-0.573	-0.173
27	NFAT1-UC1-EC	6.825	5.925	0.900	0.536	1.866	-0.900	-0.271
	NFAT1-UC2-EC	7.829	5.925	1.904	0.267	3.741	-1.904	-0.573
	NFAT1-SM-EC	7.163	5.925	1.238	0.424	2.359	-1.238	-0.373
	NFAT1-UC1-NEC	6.825	5.912	0.913	0.531	1.882	-0.913	-0.275
	NFAT1-UC2-NEC	7.829	5.912	1.916	0.265	3.774	-1.916	-0.577
	NFAT1-SM-NEC	7.163	5.912	1.251	0.420	2.379	-1.251	-0.376
28	ETS1-UC1-EC	5.789	5.015	0.774	0.585	1.710	-0.774	-0.233
	ETS1-UC2-EC	6.509	5.015	1.493	0.355	2.816	-1.493	-0.450
	ETS1-SM-EC	5.781	5.015	0.765	0.588	1.700	-0.765	-0.230
	ETS1-UC1-NEC	5.789	5.024	0.765	0.588	1.699	-0.765	-0.230
	ETS1-UC2-NEC	6.509	5.024	1.485	0.357	2.799	-1.485	-0.447
	ETS1-SM-NEC	5.781	5.024	0.757	0.592	1.690	-0.757	-0.228
29	cMAF-UC1-EC	6.499	6.788	-0.289	1.222	NA	0.289	0.087
	cMAF-UC2-EC	7.283	6.788	0.496	0.709	1.410	-0.496	-0.149
	cMAF-SM-EC	6.485	6.788	-0.303	1.233	NA	0.303	0.091
	cMAF-UC1-NEC	6.499	6.676	-0.177	1.131	NA	0.177	0.053
	cMAF-UC2-NEC	7.283	6.676	0.607	0.656	1.523	-0.607	-0.183
	cMAF-SM-NEC	6.485	6.676	-0.191	1.141	NA	0.191	0.057
30	cJUN-UC1-EC	7.720	8.066	-0.346	1.271	NA	0.346	0.104
	cJUN-UC2-EC	8.623	8.066	0.556	0.680	1.471	-0.556	-0.167
	cJUN-SM-EC	6.990	8.066	-1.076	2.108	NA	1.076	0.324
	cJUN-UC1-NEC	7.720	8.156	-0.436	1.353	NA	0.436	0.131
	cJUN-UC2-NEC	8.623	8.156	0.467	0.724	1.382	-0.467	-0.141
	cJUN-SM-NEC	6.990	8.156	-1.166	2.244	NA	1.166	0.351
31	SOCS1-UC1-EC	6.314	8.327	-2.013	4.036	NA	2.013	0.606
	SOCS1-UC2-EC	6.953	8.327	-1.374	2.592	NA	1.374	0.414

	SOCS1-SM-EC	6.141	8.327	-2.186	4.551	NA	2.186	0.658
	SOCS1-UC1-NEC	6.314	8.666	-2.352	5.104	NA	2.352	0.708
	SOCS1-UC2-NEC	6.953	8.666	-1.713	3.278	NA	1.713	0.516
	SOCS1-SM-NEC	6.141	8.666	-2.525	5.755	NA	2.525	0.760
32	SOCS3-UC1-EC	5.897	6.808	-0.911	1.880	NA	0.911	0.274
	SOCS3-UC2-EC	5.872	6.808	-0.935	1.912	NA	0.935	0.282
	SOCS3-SM-EC	5.165	6.808	-1.643	3.122	NA	1.643	0.494
	SOCS3-UC1-NEC	5.897	6.648	-0.751	1.683	NA	0.751	0.226
	SOCS3-UC2-NEC	5.872	6.648	-0.776	1.712	NA	0.776	0.233
	SOCS3-SM-NEC	5.165	6.648	-1.483	2.795	NA	1.483	0.446
33	p38-UC1-EC	4.552	4.731	-0.180	1.133	NA	0.180	0.054
	p38-UC2-EC	4.019	4.731	-0.713	1.639	NA	0.713	0.215
	p38-SM-EC	4.322	4.731	-0.409	1.328	NA	0.409	0.123
	p38-UC1-NEC	4.552	4.891	-0.339	1.265	NA	0.339	0.102
	p38-UC2-NEC	4.019	4.891	-0.872	1.830	NA	0.872	0.262
	p38-SM-NEC	4.322	4.891	-0.568	1.483	NA	0.568	0.171
34	BATF-UC1-EC	7.215	7.927	-0.713	1.639	NA	0.713	0.215
	BATF-UC2-EC	7.740	7.927	-0.187	1.138	NA	0.187	0.056
	BATF-SM-EC	7.283	7.927	-0.645	1.563	NA	0.645	0.194
	BATF-UC1-NEC	7.215	7.977	-0.762	1.696	NA	0.762	0.230
	BATF-UC2-NEC	7.740	7.977	-0.237	1.178	NA	0.237	0.071
	BATF-SM-NEC	7.283	7.977	-0.694	1.618	NA	0.694	0.209
35	IL8-UC1-EC	9.688	8.437	1.251	0.420	2.380	-1.251	-0.377
	IL8-UC2-EC	10.205	8.437	1.767	0.294	3.404	-1.767	-0.532
	IL8-SM-EC	8.768	8.437	0.331	0.795	1.258	-0.331	-0.100
	IL8-UC1-NEC	9.688	9.097	0.591	0.664	1.506	-0.591	-0.178
	IL8-UC2-NEC	10.205	9.097	1.108	0.464	2.155	-1.108	-0.333
	IL8-SM-NEC	8.768	9.097	-0.329	1.256	NA	0.329	0.099
36	IRF1-UC1-EC	2.938	3.431	-0.492	1.407	NA	0.492	0.148
	IRF1-UC2-EC	2.881	3.431	-0.550	1.464	NA	0.550	0.165
	IRF1-SM-EC	2.932	3.431	-0.499	1.413	NA	0.499	0.150
	IRF1-UC1-NEC	2.938	3.641	-0.702	1.627	NA	0.702	0.211
	IRF1-UC2-NEC	2.881	3.641	-0.760	1.693	NA	0.760	0.229
	IRF1-SM-NEC	2.932	3.641	-0.709	1.635	NA	0.709	0.213
37	RANTES-UC1-EC	4.096	3.179	0.918	0.529	1.889	-0.918	-0.276
	RANTES-UC2-EC	5.073	3.179	1.894	0.269	3.717	-1.894	-0.570
	RANTES-SM-EC	4.802	3.179	1.624	0.324	3.082	-1.624	-0.489
	RANTES-UC1-NEC	4.096	3.114	0.982	0.506	1.975	-0.982	-0.296
	RANTES-UC2-NEC	5.073	3.114	1.959	0.257	3.887	-1.959	-0.590
	RANTES-SM-NEC	4.802	3.114	1.688	0.310	3.223	-1.688	-0.508
38	BCL6-UC1-EC	4.315	4.258	0.057	0.961	1.040	-0.057	-0.017
	BCL6-UC2-EC	3.675	4.258	-0.583	1.498	NA	0.583	0.176
	BCL6-SM-EC	3.965	4.258	-0.293	1.226	NA	0.293	0.088
	BCL6-UC1-NEC	4.315	4.550	-0.234	1.176	NA	0.234	0.071
	BCL6-UC2-NEC	3.675	4.550	-0.874	1.833	NA	0.874	0.263
	BCL6-SM-NEC	3.965	4.550	-0.585	1.500	NA	0.585	0.176
39	PD1-UC1-EC	12.354	14.330	-1.977	3.936	NA	1.977	0.595

PD1-UC2-EC	13.788	14.330	-0.542	1.456	NA	0.542	0.163
PD1-SM-EC	13.203	14.330	-1.128	2.185	NA	1.128	0.340
PD1-UC1-NEC	12.354	13.999	-1.645	3.128	NA	1.645	0.495
PD1-UC2-NEC	13.788	13.999	-0.211	1.157	NA	0.211	0.063
PD1-SM-NEC	13.203	13.999	-0.796	1.737	NA	0.796	0.240

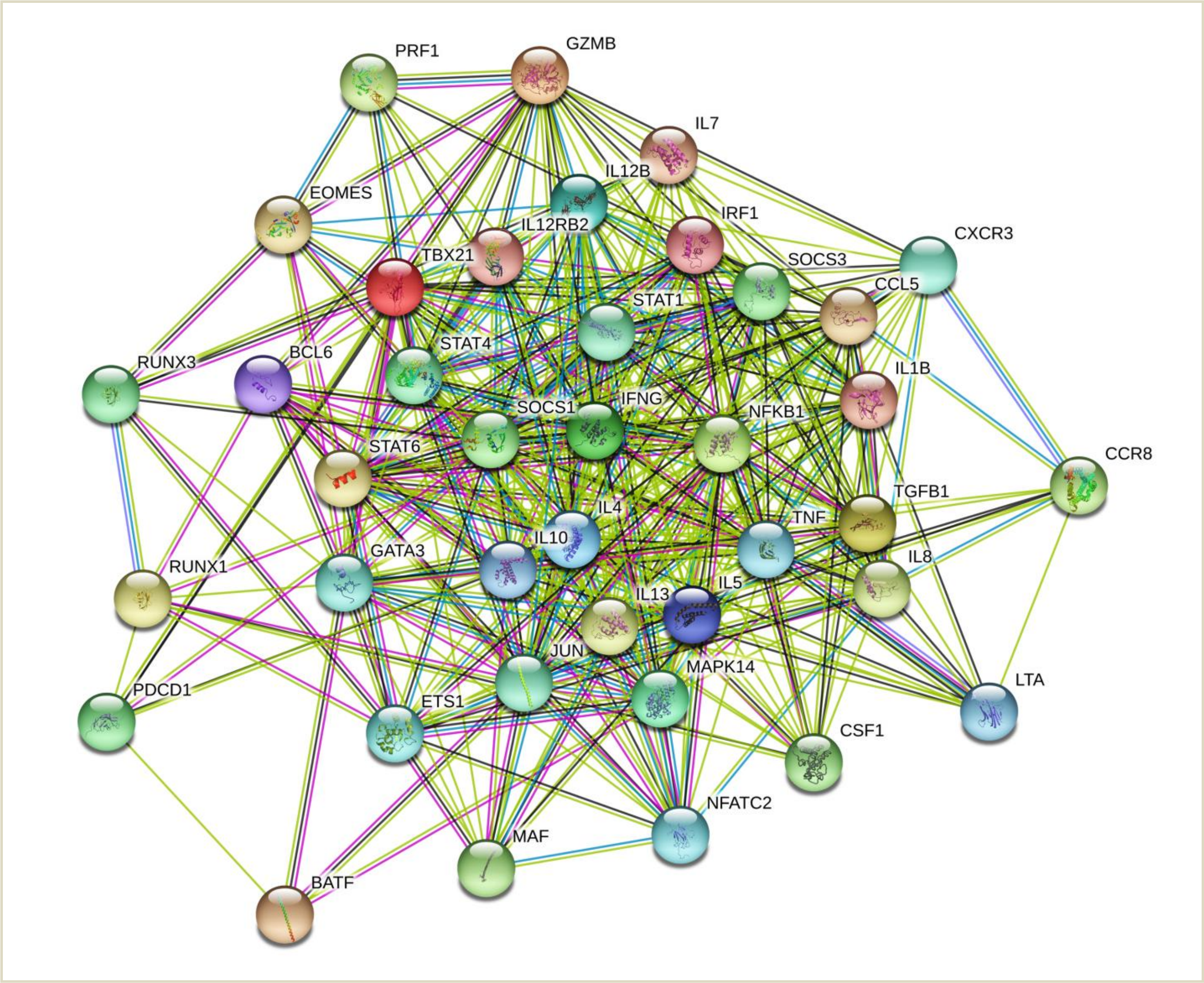

	Represents curated database	Interactions are known
	Represents experimentally determined	
	Represents neighborhood genes	Interactions are predicted
	Represents gene fusions	
	Denotes genes co-occurrence	
	Obtained by textmining	Others
	Co-expression factors	
	Homology proteins	

Figure S1. Above protein-protein interaction network was prepared using 'STRING' database (https://string-db.org/) and represented for all the 39 cytokines and regulatory factors. This interaction illustrate how these cytokines and their regulatory signaling/ transcription factors coordinates with each other and construct a complex functional network. Though some of them might physically interact with the other proteins, they can indirectly influence the expression of others or together contribute to a shared functionality. These molecules expressions and regulations usually go through alterations upon any disease burden and thus modify the interaction pattern of the whole co-ordination complex for that and/or majority of the factors.
